# Medicine Men of the World

**Published:** 1888-06-16

**Authors:** 


### Medicine Men of the World.

By the Man in the Crowd.

(Continued from p. 394, vol. iii.)

IX.

In a previous article allusion was made to the difficulty sometimes experienced by medicine men, conjurors, rain-makers, and mystery-men generally in getting out of their professions and retiring from practice. Dr. Livingstone has recorded a difficulty somewhat similar in its nature in which he once found himself.

Africans generally have a strong sense of impropriety in men having anything to do with obstetrical matters; but, says Dr. Livingstone, "a case of twins happening, and the ointments of all the doctors of the town proving utterly insufficient to effect the relief which a few seconds of English art afforded, the prejudice vanished at once." After that, he goes on to explain, he often had the satisfaction of conferring great benefits on poor women in their hour of sorrow. He was obliged, however, to confine himself to difficult cases, or he would have done nothing else; and his skill in this line brought him such fame that he had the greatest difficulty in convincing the people that his powers were not far more extensive than they actually were. A woman came to him a distance of a hundred miles seeking relief from a complaint which seemed to have baffled the native doctors. He completely cured her, and twelve months after she gave birth to a son, putting an end to her husband's reproaches for her barrenness. She sent the doctor a handsome present, and proclaimed all over the district that he possessed a medicine for the cure of sterility. "The consequence was," writes Livingstone, "that I was teased with applications from husbands and wives from all parts of the country. Some came upwards of 200 miles to purchase the great boon, and it was in vain for me to explain that I had only cured a disease in the other case. The more I denied, the higher the offers rose; they would give any money for the 'child medicine.'"

This, and a good many other stories of the kind, may suggest perhaps, that among the noble savage there are unlimited fees and rewards for successful practitioners; and that a well-equipped white man going out into some of the odd corners of the earth may make a fortune a good deal sooner than he is likely to do under ordinary circumstances in England. There are, however, difficulties in the way of attaining this, not the least of which is the peril which a white doctor may incur from his savage patient if he does not effect a satisfactory cure, and from the rival savage doctor if he does. Livingstone himself had to be very careful in his attitude towards the native medicine men. He never criticised their proceedings before others or in any way presumed to teach them in public. But he would take them aside, and give them in private useful hints and information, for which they were always very grateful, though if such instruction had been tendered before their people it would naturally have been felt to be humiliating, and would have engendered ill-feeling towards him. Only when the native doctors had given a case up, and themselves appealed to him, would he interfere with their treatment of patients, and thus he was enabled to continue on excellent terms with the medicine men, among whom he found the knowledge of many valuable medicines, and not infrequently a considerable degree of sound medical practice, based on many generations of study and observation.

But another difficulty that would stand in the way of the European doctor who should go out into savage lands to make his fortune would consist in the fact that his fees would necessarily be paid in kind, and it might be rather embarrassing "kind." Livingstone, at the time he took the honorary degree conferred upon him by Oxford University gave a lecture on his African experience in the Sheldonian Theatre, and convulsed his learned and dignified audience by a story showing the awkward form in which rewards sometimes came to him. A chief, on one occasion, sent him as a present a girl of about fifteen, if we remember right. Livingstone, of course, sent her back with an intimation that he could not think of accepting such a gift. The chief concluded that the young girl was not exactly to the white man's liking, "and," concluded the newly-made doctor, amid universal laughter, "he sent me a bigger one." Dr. Moffat gives an interesting insight into the forms which medical fees are wont to take in Africa, and the way in which the medicine-man secures them. "In many instances the slaughtering of animals on the occasion of a tree being struck by lightning, or to procure rain, or to restore the sick, may be easily traced to the inventive brain of wily rain-makers, who, in such a case, as at their public festivals and ceremonies, never neglect their stomachs. One will try to coax the sickness out of a chieftain by setting him astride of an ox which has its feet and legs tied, and then smothering the animal by holding its nose in a large bowl of water. A feast follows, and the ox is devoured, sickness and all. A sorcerer will pretend he cannot find out a guilty person, or where a patient's malady lies till he has got him to kill an ox." When the slaughter has been effected, and the good doctor gets the rump steak or whatever little tit-bit he may have a preference for, he begins to understand the matter, and the slaughter of something else the next day will probably be "indicated," as doctors say. "One will require a goat, which he kills over the sick person, allowing the blood to run down the body; another will require the fat of the kidney of a fresh-slaughtered sheep, saying that any old fat will not do; and then he gets his chop. These slaughterings are prescribed according to the wealth of the individual, so that a stout ox might be a cure for a slight cold in a chieftain, while a kid might be a remedy for a fever among the poor, from whom there was no chance of anything greater."

The traveller Bruce has told a story which, if it may be relied upon, seems to show that there are parts of the world where a physician, however skilful and however fashionable his practice, would find it exceedingly hard to grow rich. An important personage once came to him to be cured of an intermittent fever. The sufferer came to stay with the medical man he had chosen, and brought with him two servants to be quartered on him also. As, however, the great man who thus came to do honour to the stranger was very well able to smooth the way through hostile tribes to the head of the Nile, or the region in which Bruce hoped to find the head of the Nile, he gladly took him in and doctored him. When he had cured the patient and the time had come when it seemed desirable that they should part, Bruce gave the great man a hint. He replied that he was perfectly ready to go as soon as his medical benefactor should give him his clothes. Bruce did not understand this, but supposed the dusky chief must be alluding to some parcel of garments he had brought with him for use when he had recovered. He found, however, that his patient had nothing but what he had upon his person; and when the great traveller in some perplexity gently alluded to the subject, he was plainly told that his guest would rather stay where he was all his life than be so disgraced as to leave it after so long a stay without a complete new suit. "What signifies your curing me," asked the convalescent chieftain, "if you turn me out of your house like a beggar?" Whether all this was a jest, Bruce could not make out, and he laughingly went to a native friend for enlightenment. "There is no doubt," said the friend, "that you must clothe him; it is the custom." "And his servants too?" inquired Bruce. "Certainly, his servants too. If he had ten servants that ate and drank in your house, you must clothe them all." Bruce intimated that it would appear to be a point of policy on the part of a physician to let his patients die, rather than cure them at his own expense, and have to clothe them afterwards. It was explained, however, that the chief was rich, and that the clothing was required merely to show to the world how highly he was respected. He would be sure to make some kind of return. It was the invariable custom, and if the traveller wished to pass for a man of consequence, and not to have his recovered patient for his enemy, he must certainly comply with it.

